# Photons, phonons, and plasmons with orbital angular momentum in plasmas

**DOI:** 10.1038/srep41731

**Published:** 2017-02-06

**Authors:** Qiang Chen, Hong Qin, Jian Liu

**Affiliations:** 1 School of Nuclear Science and Technology and Department of Modern Physics, University of Science and Technology of China, Hefei, Anhui 230026, China; 2 Luoyang Electronic Equipment Testing Center, Luoyang 471000, China; 3 Plasma Physics Laboratory, Princeton University, Princeton, NJ 08543, USA

## Abstract

Exact eigen modes with orbital angular momentum (OAM) in the complex media of unmagnetized homogeneous plasmas are studied. Three exact eigen modes with OAM are derived, i.e., photons, phonons, and plasmons. The OAM of different plasma components are closely related to the charge polarities. For photons, the OAM of electrons and ions are of the same magnitude but opposite direction, and the total OAM is carried by the field. For the phonons and plasmons, their OAM are carried by the electrons and ions. The OAM modes in plasmas and their characteristics can be explored for potential applications in plasma physics and accelerator physics.

During the last quarter century, the generation, transmission, conversion and detection techniques of photon orbital angular momentum (OAM) experienced significant advances, due to their wide applications in quantum information, particle manipulation, non-classical imaging, nanotechnology and astronomy^1^,^2^,^3^,^4^,^5^,^6^,^7^,^8^,^9^,^10^,^11^,^12^,^13^,^14^,^15^,^16^,^17^,^18^. In 1990, Tamm *et al*. generated Laguerre-Gaussian (LG) mode laser beams which have helical wave fronts and can drive neutral atoms and molecules^1^. Allen *et al*. first demonstrated that light beams with an azimuthal phase distribution carries an angular momentum independent of the polarization photon state^2^. The lost part of photon angular momentum embedded in twisted electromagnetic beam or optical vortex was found. Recently, the extension of photon OAM technology from visible to radio frequencies and the discovery of efficient OAM modes generation and sorting methods lead to more potential application in science and engineering^8^,^11^,^14^,^19^. Most of the previous studies are based on paraxial optics with slow varying envelope approximation in vacuum or crystal, as this approximation is valid in most experimental conditions. Recently, the LG modes have been corroborated by particle-in-cell (PIC) simulations of intense laser-plasma interactions^20^,^21^. Exact solutions of photons with OAM in vacuum can be obtained without invoking the paraxial approximation, which have general theoretical significance^22^.

Electromagnetic waves in plasmas and their interaction with charged particles play a crucial role in plasma physics and accelerator physics. RF waves are employed to accelerate particles in modern accelerators^23^,^24^, and to heat plasmas and drive current in magnetic fusion devices^25^. They are also the most effective plasma diagnostic tools. The coupling from injection waves to fusion plasmas can excite abundant eigen modes, such as the electron cyclotron wave, ion cyclotron wave and Bernstein wave. Different modes have different propagation properties, such as the accessibility and absorption characteristics, which determine their applicability^26^. Although this classical problem has been studied extensively with wide applications, attentions have been paid to the OAM phenomena in plasmas only in recent years^13^,^20^,^21^,^27^,^28^,^29^,^30^,^31^,^32^,^33^,^34^,^35^,^36^,^37^.

Does a plasma support exact electromagnetic or other type of eigen modes with OAM? If so, can they be utilized to achieve better diagnostics, heating, current drive, and particle acceleration? In the present study, we address these two questions. In the past, measurements of the interaction between a RF wave with OAM and plasma vortex was made, and theoretical descriptions for plasma waves with OAM based on the LG modes were given by Mendonça and collaborators^20^,^21^,^27^,^28^,^29^,^30^,^31^,^32^,^33^,^34^,^35^,^36^,^37^,^38^. Also using the LG modes, electromagnetic and electrostatic waves in the fluid model^27^,^28^,^29^,^30^,^31^,^32^, inverse Faraday effect^38^, kinetic description^33^,^34^ and Landau damping for twisted waves were studied^35^,^36^. Additionally, Shukla studied the twisted shear Alfvén waves^13^. However, LG modes are solutions under the scalar paraxial approximation assuming a slowly varying envelope. They are not rigorous solutions of the vector Maxwell equations. Especially, in the complex media of plasmas, detailed and careful analysis should be performed on the vector Maxwell equation with a proper self-consistent model for plasmas. In this paper, we adopt a two-fluid system which self-consistently couples the dynamics of electrons and ions with the vector Maxwell equations. We describe three classes of rigorous solutions of the system that can be identified as photons, phonons, and plasmons with OAM. They correspond to the electromagnetic, ion acoustic, and Langmuir waves in plasmas. The OAM of different plasma components are closely related to the charge polarities. For photons, the OAM of electrons and ions are of the same magnitude but opposite direction, and the total OAM is carried by the field. For phonons and plasmons, all OAM are carried by the electrons and ions. When the thermal effects are considered, there is a non-zero global OAM in general. Based on their spectrum modulation, power concentration structure and rotation properties, the OAM eigen modes have important potential applications in plasma diagnostics, heating, current drive in magnetic fusion devices and driving rotating charged particle beams with enhanced stability in high-intensity accelerators.

## Physical model

To study the small amplitude electromagnetic waves in a plasma, we start from a linearized two-fluid system with self-consistent electromagnetic field determined by the Maxwell equations,


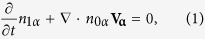



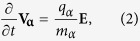



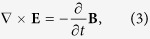



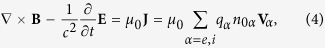



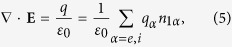



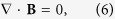


where the subscript *α* denotes electron (e) or ion (i) component, (**E**, **B**, **V**_*α*_, *n*_1*α*_) represent the first order perturbed fields, *n*_0*α*_ is the equilibrium density, and other variables have their usual meanings. The equilibrium is assumed to be cold, homogeneous, unmagnetized, zero flow and satisfies the neutrality condition ∑_*α*=*e*,*i*_*q*_*α*_*n*_0*α*_ = 0. The thermal effects will be considered in the second half of the paper.

The linear system (1)–(6) admits two approximations. The first is the electromagnetic approximation where the quasi-neutrality condition ∑_*α*=*e*,*i*_*q*_*α*_*n*_1*α*_ = 0 is assumed and the system is reduced to


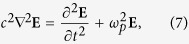



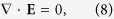


where 
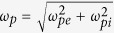
 and 
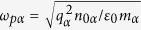
. The second is the electrostatic approximation where the perturbed magnetic field is negligible and the system is reduced to


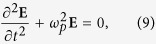



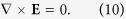


It turns out that a monochromatic mode with OAM can be constructed in the cylindrical coordinates with azimuthal phase distribution e^*ilϕ*^, where the integer *l* is the azimuthal mode number. In quantum optics, it is known as topological charge describing the degree of phase helicity^12^. The mode assumes the form of


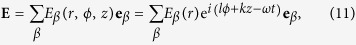


where the subscript *β* = *r, ϕ*, or *z* denotes cylindrical coordinates, and *E*_*β*_(*r*) is a function of the radial coordinate. The *z*–direction is the space-time averaged propagation axis.

## Photons with OAM in plasmas

We first look at the electromagnetic modes, i.e., photons, with OAM. Substituting Eq. (11) into Eq. (7), we obtain the eigen equation of the electromagnetic modes,


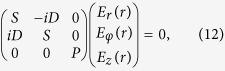


where *D* = 2*l*/*r*^2^, and other matrix elements are defined as,


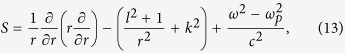



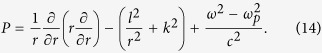


In terms of *E*^±^(*r*) ≡ *E*_*r*_(*r*) ± *iE*_*ϕ*_(*r*), Eq. (12) can be rewritten as


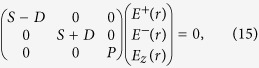


which shows that the *E*^+^(*r*), *E*^−^(*r*), and *E*_*z*_(*r*) components are decoupled. These components are analogues of the angular momentum operators *L*_±_ and *L*_*z*_ in quantum mechanics. A class of special solutions of eigen equation which satisfies the finite boundary condition at axis are 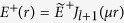, 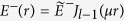 and 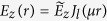, where *J*_*l*_(*μr*) is the *l*–th order Bessel function, and 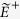, 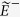 and 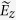 are undetermined constants. Here, *μ* is a constant that can be viewed as a special kind of wave number in the *r*-direction, because any function *f*(*r*) defined on the domain of 0 ≤ *r* ≤ *R* can be expanded using the *l*–th order Bessel function *J*_*l*_ but with different values of *μ*. This expansion is of course the familiar Bessel-Fourier expansion *f*(*r*) = ∑_*n*_*c*_*n*_*J*_*l*_(*μ*_*n*_*r*), where *Rμ*_*n*_ is the *n*–th positive root of *J*_*l*_(*r*) and *c*_*n*_ is an expansion coefficient determined by *f*(*r*). As discussed by Barnett *et al*.^22^, the paraxial waves, e.g. LG modes, can be obtained by Bessel mode superposition. For the LG mode 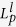, the expansion coefficient is 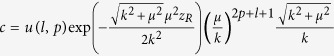, which can verified by direct substitution^22^. Here, *u*(*l, p*) is a constant and *z*_*R*_ is the Rayleigh range, and *μ* is continuously varying.

All three mode components have the same dispersion relation,


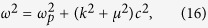


which indicates that the three modes are degenerate states. However, the divergence free condition, i.e., Eq. (8), puts on a constraint on the *E*^+^(*r*), *E*^−^(*r*), and *E*_*z*_(*r*) components,


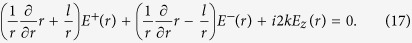


In terms of 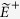, 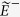 and 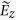, it is


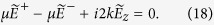


For a given pair of *k* and *μ*, the mode has two degrees of freedom or degeneracy.

The electromagnetic mode with OAM is localized around the wave axis, and the amplitude envelope decays approximately as 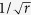 for large *r*. Because *J*_*l*_(0) = 0 when *l* ≠ 0, there is no phase singularity of photon OAM at axis. The power density of the mode maximized on a ring with a radius determined by the maximum of the *z*-component of the momentum in Eq. (21).

Here, we discuss a special case with 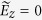 and 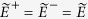. In this case,


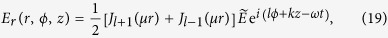



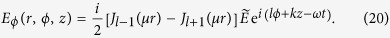


From Eqs. (19) and (20), the time averaged momentum and angular momentum densities are


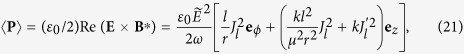



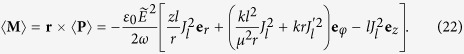


The radial component of 〈**M**〉 and the azimuthal components of 〈**P**〉 and 〈**M**〉 are symmetric about the axis, thus spatial average leaves only the *z*-components, which shows that the eigen modes carry *z*-photon OAM. From Eqs (2),(3),(4), we can also find that the OAM of electrons is opposite to that of ions, and the total OAM carried by charged particles s is zero.

Different from the scalar paraxial solutions with slow varying envelope approximation, the OAM eigen modes obtained above are rigorous analytical solutions admitted by plasmas, which are orthogonal and complete. It is not surprising to find similarities and differences between our solutions specified by Eqs. (19) and (20) and the familiar Bessel modes,


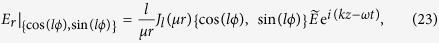



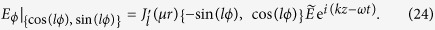


Their radial dependencies are all expressed in terms of Bessel functions, and they are both diffraction free, as there is no radial momentum component. However, there are major differences. Equations (19) and (20) give an azimuthal phase distribution, which forms a helical wave front. On the other hand, the familiar Bessel modes have two degenerate polarization components, which have orthogonal azimuthal amplitude distributions. Another important difference is that the Bessel modes carry no OAM, which can be verified by direct calculation. Interestingly, an electromagnetic mode with OAM can be constructed from two Bessel modes without OAM as


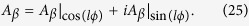


**Figure 1:**
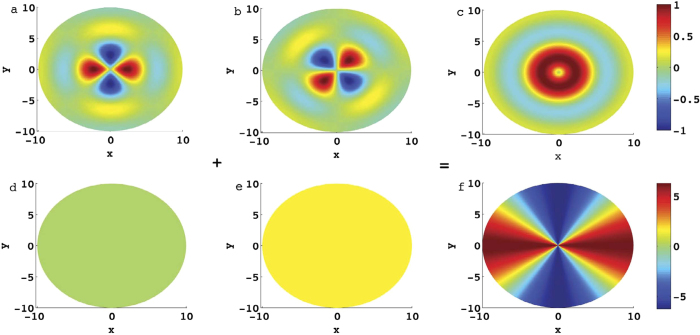
Conversion from orthogonal azimuthal amplitude distributions to a topological charge. (**a**) and (**d**) are the amplitude and phase distributions of the mode *A*_*β*_|_cos(*lϕ*)_. (**b**) and (**e**) are those of the mode *A*_*β*_|_sin(*lϕ*)_. (**c**) and (**f**) are those of the modes with OAM. The azimuthal mode number *l* is 2. The amplitudes are normalized by their maximum values and the coordinates are normalized by *μr*. The phases are measured by *rad*. It shows that a photon with OAM can be constructed from modes without OAM via the coherent interference.

Here, *A*_*β*_ denotes mode components of the electromagnetic modes with OAM obtained from Eq. (15), and *A*_*β*_|_cos(*lϕ*)_ and *A*_*β*_|_sin(*lϕ*)_ are the degenerate Bessel modes without OAM. The Euler formula *e*^*ilϕ*^ = cos(*lϕ*) + *i*sin(*lϕ*) realizes the conversion from orthogonal azimuthal amplitude distributions to a topological charge (Fig. 1). One may wonder why one OAM mode can be excited by two modes without OAM? This effect can be attributed to the familiar coherent interference. To wit, we have


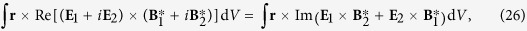


where the superscripts 1 and 2 denote two degenerate states without OAM in Eq. (25). The cross product between electric and magnetic fields of different modes leads to an azimuthal momentum distribution, and thus a twisted beam. This phenomenon is similar to the process that a circularly polarized wave with spin can be excited by two linearly polarized waves without spin.

## Plasmons and phonons with OAM in plasmas

We now investigate the electrostatic modes with OAM. Substituting Eq. (11) into Eq. (9), we obtain the electric field eigen equations of electrostatic modes with OAM. These equations can be written in a vector form as,


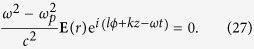


The dispersion relation obtained from Eq. (27) is that for plasma oscillation, i.e., *ω* = *ω*_*p*_, which should not be surprising. This mode can be viewed as a plasmon with OAM. The components *E*_*r*_(*r*), *E*_*ϕ*_(*r*) and *E*_*z*_(*r*) should satisfy the rotation free condition, i.e., Eq. (10),


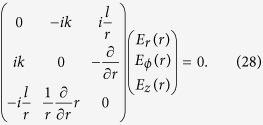


Because the rank of the coefficient matrix in Eq. (28) is 2, there are two constraints and one independent mode component. In another word, the mode is non-degenerate. Solving Eq. (28), we obtain


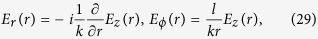


where *E*_*z*_(*r*) is an arbitrary function of *r*. However, in order to avoid phase singularity, it should satisfy the following conditions,


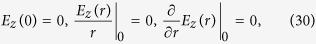


The polarization properties described by Eq. (29) show that for the electrostatic mode with OAM, the electrical field is not parallel to the space-time averaged propagation axis, which is in the *z*-direction. This situation is similar to the fact that for the electromagnetic mode with OAM, the electrical field is not perpendicular to the space-time averaged propagation axis.

We note that the electrostatic mode, or the plasmon, with OAM is a non-propagating oscillation under the cold plasma approximation. We now investigate finite temperature effects, one of which is the formation of a new propagating electrostatic mode with OAM, i.e., phonon with OAM. When the finite temperature is considered, Eq. (2) should be modified as


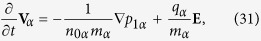


where the thermal pressures *p*_1*α*_ satisfies the polytropic law *p*_1*α*_/*p*_0*α*_ = *γ*_*α*_*n*_1*α*_/*n*_0*α*_. The thermal velocities for electron and ion are defined as 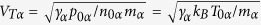, where the *γ*_*α*_ is the polytropic index. Substituting Eq. (1) into Eq. (31), we obtain,


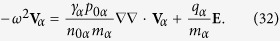


For the electromagnetic modes, Eqs (7) and (32) lead to Eq. (12), which means that there is no thermal correction for the electromagnetic modes with OAM. The thermal effect on the electrostatic modes is more interesting. It produces phonons with OAM in plasmas. With finite temperature, it is more convenient to derive the eigen system using the velocity components. Equations (9) and (32) lead to


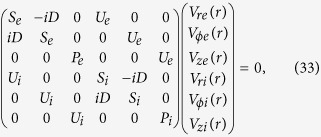


for ***V***_*α*_ = ∑_*β*_*V*_*αβ*_(*r, ϕ, z*)***e***_*β*_ = ∑_*β*_*V*_*αβ*_(*r*)e^*i*(*lϕ* + *kz*−*ωt*)^***e***_*β*_. Here, 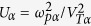, *D* was defined after Eq. (12), and *S*_*α*_ and *P*_*α*_ have similar forms as Eqs (13) and (14), except that *c*^2^ is replaced by 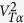 and 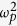 is replaced by 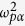, respectively.

Defining new field components 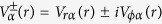, we can rewrite Eq. (33) in the principal axis system as


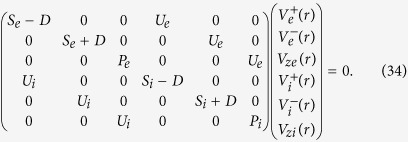


It shows that the eigen system of the electrostatic mode with OAM in a warm plasma consists of three decoupled subsystems 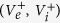, 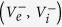 and (*V*_*ze*_,*V*_*zi*_). These subsystems have same dispersion relations, representing three degenerate states with different polarization. For the subsystem (*V*_*ze*_,*V*_*zi*_), for example, with 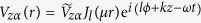, the eigen equations are,


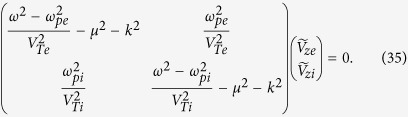


The dispersion relation given by Eq. (35) is


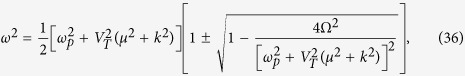



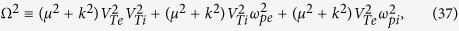


where 
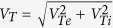
. The polarization relation between electron and ion velocities is specified by


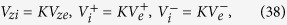


where


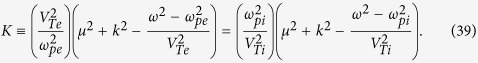


In Eq. (39), the second equal sign is another, probably more transparent, way to write the dispersion relation (36). In most cases 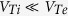, and the two branches of Eq. (36) can be simplified as


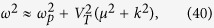



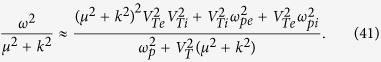


Equation (40) describes the Langmuir wave with OAM, which is a propagating plasmon with OAM. Equation (41) is the dispersion relation for the electrostatic mode which vanishes in the cold plasma limit. It is the low frequency ion acoustic wave with OAM. It can be viewed as a phonon with OAM. The OAM of the modes can be calculated from the eigen structure. Because an electrostatic mode does not carry electromagnetic momentum density^39^, the mode contains only kinetic momentum density of the particles. The first order density field of the mode is


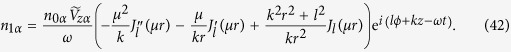


The time averaged momentum density and angular momentum density of plasma components are,


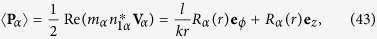



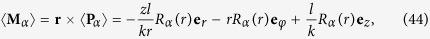


where


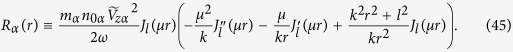


The radial component of 〈**M**_*α*_〉 and the azimuthal components of 〈**P**_*α*_〉 and 〈**M**_*α*_〉 are symmetric about the axis, and integration over space leaves only the z-components, which show that the plasma components carry z-plasmon or z-phonon OAM. Furthermore, when *m*_*e*_*n*_0*e*_ + *m*_*i*_*n*_0*i*_*K*^2^ ≠ 0, the OAM symmetry between electrons and ions are broken by the thermal effect. As a result, the mode contains a global OAM.

## Outlook

The unique properties of the OAM photons, phonons, and plasmons discussed above enable important potential applications in plasma physics and accelerator physics. As an intrinsic characteristic of the OAM beam, the highly localized power density off the propagation axis can be an effective tool for delivering focused heating and acceleration power, which can be used to heat fusion plasmas in a specified position and generate structured charged particle beams. It is also a potential plasma diagnostic technique. The OAM states can be modulated by inhomogeneous and anisotropic structures in plasmas, such as density and magnetic field fluctuations. By injecting an OAM beam and detecting the OAM scattering spectrum, we can infer statistical properties of fluctuations in the plasma. For application in accelerator physics, if electromagnetic modes with OAM are introduced as accelerating field structures, charged particle beams will be driven by the OAM of the modes to rotate. Rotating particle beams are more stable for applications where high beam intensity is required.

In this work, electromagnetic and electrostatic waves with OAM in unmagnetized homogeneous plasmas are systematically studied. Exact OAM eigen modes are derived, which are different from approximate solutions in scalar paraxial optics with slow varying envelopes. Three classes of OAM modes are discussed: photons, phonons, and plasmons, which correspond to the electromagnetic, ion acoustic, and Langmuir waves. The modes have azimuthal phase distribution and Bessel-type of radial dependency. It is found that the electromagnetic mode with OAM can be excited by two familiar Bessel modes without OAM. For the phonons and plasmons, the OAM are carried by the electrons and ions. The OAM modes in plasmas and their characteristics can be explored for various potential applications. Further studies of the propagation properties of the modes with OAM and their interactions with plasmas are expected to reveal new physics previous unknown.

## Additional Information

**How to cite this article**: Chen, Q. *et al*. Photons, phonons, and plasmons with orbital angular momentum in plasmas. *Sci. Rep.* **7**, 41731; doi: 10.1038/srep41731 (2017).

**Publisher's note:** Springer Nature remains neutral with regard to jurisdictional claims in published maps and institutional affiliations.
